# Docosahexaenoic acid and non-alcoholic fatty liver disease in obese children: a novel approach?

**DOI:** 10.1186/s12967-015-0471-7

**Published:** 2015-04-02

**Authors:** Elvira Verduci, Carlotta Lassandro, Giovanni Radaelli, Laura Soldati

**Affiliations:** Department of Pediatrics, San Paolo Hospital, University of Milan, Milan, Italy; Department of Health Sciences, University of Milan, Milan, Italy

**Keywords:** DHA, Childhood obesity, NAFLD, n-3 LCPUFA

## Abstract

Non-alcoholic fatty liver disease represents the most common chronic liver disease in obese children of industrialized countries. Nowadays the first line of treatment of pediatric non-alcoholic fatty liver disease is based on dietary and lifestyle intervention; however compliance to these interventions is very difficult to maintain in long term period. This editorial discusses about docosahexaenoic acid treatment as possible novel approach for non-alcoholic fatty liver disease in obese children. Docosahexaenoic acid may modulate the inflammatory response, improve insulin sensitivity and could be effective in enhancing intestinal barrier integrity, essential to protect a healthy gut-liver axis. Indeed alteration of gut microbiota composition and increased intestinal permeability may rise the exposure of liver to gut-derived bacterial products, causing activation of signalling pathways implicated in liver inflammation and fibrogenesis. This mechanism has been observed in vitro and animal models of non-alcoholic fatty liver disease but also in a clinical study in adults. While evidence suggests that n-3 long-chain polyunsaturated fatty acids supplementation may decrease liver fat in adults, in pediatric population only a study examined this topic. In obese children with non-alcoholic fatty liver disease well designed randomized controlled trials are needed to better clarify the possible efficacy of docosahexaenoic acid treatment, and underlying mechanisms, to identify the optimal required dose and to evaluate if the docosahexaenoic acid effect is limited to the duration of the treatment or it may continue after the end of treatment.

## Background

Non-alcoholic fatty liver disease (NAFLD), considered as liver manifestation of metabolic syndrome, represents the most common chronic liver disease in obese children of industrialized countries, with a reported prevalence of 3% to 10% in the general pediatric population and reaching a prevalence of 80% in obese or overweight children [1]. NAFLD is characterised by the pathological accumulation of liver fat without relation to alcohol intake, ranging from ‘simple’ liver steatosis to non-alcoholic steatohepatitis (NASH). Significant complications of NAFLD are represented by progression to liver fibrosis and cirrhosis [2]. Excess food intake and sedentary lifestyle, resulting in obesity and insulin resistance, are important environmental risk factors associated with NAFLD [1]. However the complete pathogenesis remains unexplained and seems to involve several factors. Recently, a great deal of attention has been focused on the gut-liver axis malfunction [small intestinal bacterial overgrowth (SIBO), intestinal dysbiosis, and increased intestinal permeability (“leaky gut”)], considered as another key factor involved in development and progression of NAFLD [2]. Indeed intestinal epithelium, gut microbiota and dietary pattern are linked in different ways. For example, a high fat diet can stimulate a proinflammatory microbiota and interfere with intestinal permeability [2]. Alteration of gut microbiota composition and increased intestinal permeability may rise the exposure of the liver to gut-derived bacterial products, as lipopolysaccharides (LPS), which could determine endotoxemia. Then, endotoxemia can stimulate Toll-Like Receptors (TLR), causing activation of signalling pathways implicated in liver inflammation and fibrogenesis [3]. This mechanism has been observed in vitro and animal models of NAFLD but also a clinical study has shown that NAFLD in adults is associated with increased intestinal permeability and SIBO, related to severity of liver steatosis [4]. Moreover, Giorgio et al. [3] showed that intestinal permeability is increased also in pediatric population with NAFLD, according to severity of liver disease, suggesting its important role in NAFLD progression. Therefore it appears that intestinal barrier integrity is essential to protect a healthy gut-liver axis.

Nowadays the first line of treatment of NAFLD in obese children is represented by dietary and lifestyle intervention; however compliance to these interventions is very difficult to maintain in long term period, especially in pediatric population [1]. The aim of this editorial is to discuss the possible role of DHA in treatment of NAFLD in childhood obesity with respect to current evidence.

## Main text

Several studies showed that NAFLD is characterized by a low total level of n-3 fatty acids, in turn associated with steatosis, increased oxidative stress and NASH [5]. Moreover, from studies in NAFLD experimental models, it has been shown that n-3 fatty acids may alter liver gene expression (switching intracellular metabolism from lipogenesis and storage to fatty acid oxidation and catabolism), improve insulin sensitivity, have anti-inflammatory properties and reduce tumor necrosis factor levels [5].

Supplementation with n-3 long-chain polyunsaturated fatty acids (LCPUFA), and in particular DHA, has been experienced as potential treatment for obesity-related NAFLD especially in adult population. Indeed, a recent systematic review showed that n-3 LCPUFA (eicosapentaenoic acid and docosahexaenoic acid) supplementation may decrease liver fat in adults [6]. Currently, there is in literature only a study examining this topic in pediatric age [7]. Indeed a randomized controlled trial reported that DHA supplementation was associated with improved liver steatosis in obese children with NAFLD [7]. This study showed that DHA supplementation may reduce liver hyperechogenicity in children with NAFLD after 6 months of treatment, with comparable effect using a dose of 250 mg/day or 500 mg/day of DHA. The improvement in echogenicity observed at 6 months remained unchanged also after 24 months of treatment. Hovewer, it should be pointed out that in this study the results were obtained using liver ultrasound sonography test (US) only and caution has to be paid in inferring any definitive conclusion. Indeed Chemical shift magnetic resonance imaging (MRI) (opposed-phase imaging) should be also considered for its recognized its ability to quantify hepatic fat content accurately, and in turn to identify fat regression or accumulation over time in children with NAFLD [8,9].

The potential protective effect of DHA has been suggested also from a retrospective study evaluating early type of feeding (breastfed versus formula-fed and duration of breastfeeding) in a cohort of children with NAFLD [10]. It has been speculated that DHA, delivered by human milk after prolonged lactation, could be protective against progression of the liver disease from simple steatosis to steatohepatitis and fibrosis (NASH) acting as peroxisome proliferator-activated receptors agonist (PPAR-agonist), transcription factor involved in protection against fibrosis [11]. Recently it has been also suggested that DHA, and in particular two active metabolites derived from it, resolvins and protectins, can modulate the inflammatory response not only by decreasing cytokine production but also with promotion of the resolution of inflammation [12]. Indeed, animal models showed that these mediators might reduce inflammation induced by excessive adipose tissue and improve insulin resistance, stimulating adiponectin expression [12].

Furthermore, considering the importance of the gut-liver axis in the development and progression of NAFLD, a significant result, derived from animal models, suggests that DHA could be effective in enhancing intestinal barrier integrity, by increasing, for example, protein expression of intestinal tight junction proteins [12]. It seems to be a bi-directional relationship between DHA and gut microbiota: DHA may alter the gut microbial populations, and some microbial species such as *Bifidobacterium* may improve the tissue distribution of DHA [12].

## Conclusions

In conclusion, DHA might exert a positive role in treatment of NAFLD in pediatric age but it remains to be demonstrated. Further well designed randomized controlled trials are needed to better clarify the possible efficacy of DHA treatment, and underlying mechanisms, in obese children with NAFLD, to identify the optimal required dose and to evaluate if the DHA effect is limited to the duration of the treatment or it may continue after the end of treatment.

## Abbreviations

DHA: Docosahexaenoic acid
LCPUFA: Long-chain polyunsaturated fatty acids
LPS: Lipopolysaccharides
MRI: Magnetic resonance imaging
NAFLD: Non-alcoholic fatty liver disease
NASH: Non-alcoholic steatohepatitis
PPAR-agonist: Peroxisome proliferator-activated receptors agonist
SIBO: Small intestinal bacterial overgrowth
TLR: Toll-Like Receptors
US: Ultrasound sonography
